# Anti-inflammatory Effect of a Novel Pectin Polysaccharide From *Rubus chingii* Hu on Colitis Mice

**DOI:** 10.3389/fnut.2022.868657

**Published:** 2022-04-29

**Authors:** Yuanfang Kong, Yulong Hu, Jieming Li, Juntao Cai, Yuanhao Qiu, Chunhong Dong

**Affiliations:** ^1^College of Pharmacy, Henan University of Chinese Medicine, Zhengzhou, China; ^2^Academy of Chinese Medical Science, Henan University of Chinese Medicine, Zhengzhou, China; ^3^Henan Polysaccharide Research Center, Zhengzhou, China; ^4^Henan Key Laboratory of Chinese Medicine for Polysaccharides and Drugs Research, Zhengzhou, China; ^5^College of Medicine, Pingdingshan University, Pingdingshan, China

**Keywords:** *Rubus chingii* Hu polysaccharide, separation and purification, structural characterization, anti-inflammatory activity, inflammatory bowel disease

## Abstract

*Rubus chingii* Hu has been used as a functional food for a long time. A novel pectin polysaccharide named RCHP-S from *R. chingii* Hu was structurally identified and explored its anti-inflammatory effect on colitis mice. RCHP-S was composed of mannose, rhamnose, glucuronic acid, galacturonic acid, glucose, galactose, and arabinose. NMR spectroscopy and methylation analysis showed that RCHP-S was mainly composed of HG-type pectin domains but also contains a small amount of RG-I. The anti-inflammatory tests indicated that the mouse macrophage RAW 264.7 cells pretreated with RCHP-S could show a significant inhibitory effect on the mRNA level of iNOS, IL-1β, IL-6, and TNF-α *in vitro*. Polysaccharide RCHP-S reduced the enteritis symptoms in dextran sulfate sodium (DSS)-induced colitis mice by inhibiting released inflammatory factors. These results indicated that the *R. chingii* Hu polysaccharide can be used as food additives for the treatment of intestinal inflammation.

## Introduction

Inflammatory bowel disease (IBD), including Crohn's disease (CD) and ulcerative colitis (UC), is a chronic inflammatory disease of intestinal mucosa caused by multiple factors in genetically susceptible individuals (1). UC mainly occurs in the colonic mucosa and submucosa, which exhibit abdominal pain, diarrhea, mucus-like bloody stool, etc. Recurrence and remission occur alternately of intestinal inflammation in clinic (2). During the continued inflammatory process, it has been reported that lots of inflammatory factors (such as TNF-α, IL-1β, IL-6, and IL-8) have increased expression (3). So, it is important to develop drugs that have anti-inflammatory factors for UC.

Polysaccharides have a variety of important biological activities and are widely distributed in the animal cell membrane, plant cell walls, and microorganisms, so they have attracted more and more attention from researchers (4). Polysaccharides are used as ideal natural anti-inflammatory drugs (5), such as the natural polysaccharides (6), *longan pulp* polysaccharide LPIIa (7), an inulin-type fructan, (8) etc., which had anti-inflammatory properties. Combined, the anti-inflammatory activity of polysaccharides has attracted increasing attention.

*Rubus chingii* Hu is a plant belonging to the Rosaceae family and is extensively grown in many regions of the world, including Europe, Asia, and North America. It is identified as “Fu-pen-zi” in Chinese and has been used for a long time as a healthy food in China (9). *R. chingii* Hu has various biological functions as a medicine, including flavonoid glycosides due to which fruits display anti-inflammatory activity by suppressing the activation of MAPKs in macrophages (10). However, few studies have explored polysaccharides in *R. chingii* Hu. Only crude polysaccharides from the fruits and leaves of *R. chingii* Hu were explored for their bioactivities and extraction optimization (11) and new functions of polysaccharides from *R. chingii* Hu, which has protective effects against ethyl carbamate-induced cytotoxicity, were studied (12).

At present, there are few reports on the anti-inflammatory activity of polysaccharides from *R. chingii* Hu. A novel purified polysaccharide named RCHP-S was obtained from *R. chingii* Hu and purified by column chromatography and its characteristic structures were analyzed in this study. In addition, the anti-inflammation of RCHP-S in lipopolysaccharide (LPS) stimulated the mouse macrophage RAW 264.7 cells and dextran sulfate sodium (13) (DSS)-induced colitis mice were also explored. This work provides an experimental basis for *R. chingii* Hu polysaccharides as a food additive to reduce intestinal inflammation.

## Materials and Methods

### Materials

The chemical reagents were all of the analytical grades. *R. chingii* Hu was obtained from Guangxi province in China. The reference standards of glucose, galactose, rhamnose, xylose, arabinose, fucose, mannose, glucuronic acid, galacturonic acid, 1-phenyl-3-methyl-5-pyrazolone (PMP), trifluoroacetic acid (TFA), and lipopolysaccharide (LPS) were purchased from Sigma–Aldrich (St. Louis, CA, USA). Sulfasalazine (SSZ) and dextran sulfate sodium (DSS) were purchased from Aladdin (Shanghai, China). Mouse interleukin (IL)-1β and IL-6 enzyme-linked immunosorbent assay (ELISA) kits were purchased from Enzyme-linked Biotechnology Corporation (Shanghai, China).

### Extraction of *R. chingii* Hu Crude Polysaccharides

After degreasing with 95% alcohol, fresh *R. chingii* Hu was extracted with ten-fold distilled water at 80°C for 4 h. Then, the extracts were concentrated. A four-fold amount of absolute ethyl alcohol was slowly added to the concentrated solution, stirred uniformly, and alcohol-precipitated overnight. The precipitate was collected and dialyzed (3,500 Da) with distilled water. The dialysate was further concentrated by a rotary evaporator and then freeze-dried to obtain crude polysaccharides of *R. chingii* Hu.

### Purification of *R. chingii* Hu Crude Polysaccharides and Molecular Weights

An appropriate amount of crude polysaccharides of *R. chingii* Hu need to be further purified by DEAE cellulose-52 column (6 × 50 cm) and gel column chromatography. Distilled water, 0.1 M NaCl, 0.2 M NaCl, and 0.5 M NaOH, were used as eluents. The same elution fraction was combined and concentrated to detect saccharide concentration with the phenol–H_2_SO_4_ method. The polysaccharides obtained using a DEAE cellulose-52 column were further purified by gel column chromatography. The fully swollen gel filler G-25 Sephadex and Sephacryl S300 were loaded into two slender chromatography columns (2.5 × 60 cm). The polysaccharide sample purified by DEAE-52 column chromatography was dissolved in 0.2 M NaCl, the sample was loaded slowly, and 0.2 M sodium chloride solution was then used for washing, after which homogeneous polysaccharides named RCHP-S were obtained after freeze-drying the fractions.

The high-performance gel permeation chromatography (HPGPC) was used to detect the purity and molecular weight of polysaccharide samples, which were connected to three serially linked ultrahydrogel columns (250, 1,000, and 2,000, 300 mm × 7.8 mm, 6 μm particles). The oven temperature was chosen as 40°C. T-Dextrans (5.2 × 10^3^, 48.6 × 10^3^, 668 × 10^3^ Da) were set as the standards. The standard curve is log (Mw) = 12.1317 – 0.1863T.

### Characteristic Structure Analysis of RCHP-S

The neutral sugar content of polysaccharides was determined by the sulfuric acid phenol method, using glucose as the standard (14). The uronic acid content of polysaccharides was determined by the meta-hydroxybiphenyl method, and galacturonic acid was used as the standard (15). The protein content of polysaccharides was determined by the Coomassie brilliant blue, using globulin as the standard (16).

### Monosaccharide Compositions

The monosaccharide composition of RCHP-S was analyzed with the 1-phenyl-3-methyl-5-pyrazolone (PMP) pre-column derivatization method by using high performance liquid chromatography (HPLC). The polysaccharide RCHP-S was hydrolyzed with 2 M TFA at 110°C for 4 h, then derivatized with PMP. The monosaccharide standards and the various monosaccharide compositions were examined *via* HPLC (NU3000, Hanbon Sci & Tech, China). The UV detection wavelength was set at 254 nm. A mobile phase with 80% of 0.05 M KH_2_PO_4_ (pH = 6.9) and 20% of acetonitrile mixture was used with a flow rate of 1.0 ml/min (17).

### NMR Analysis

The 20 mg of the polysaccharide sample was dissolved in D_2_O. After lyophilization, it will be re-dissolved in 500 μl of deuterated water. The ^1^H NMR, ^13^C NMR, and two-dimensional (^1^H-^1^H COSY, ^1^H-^13^C HSQC, ^1^H-^13^C HMBC) scanning analysis of *R. chingii* Hu polysaccharides were completed using the Bruker AM 400 nuclear magnetic resonance (NMR) instrument at room temperature.

### Methylation Analysis

The methylation analysis was carried out according to the previously described method with slight modifications (18). The methylated fragments were hydrolyzed, reduced, and the final products were analyzed by gas chromatography–mass spectrometry (GC–MS).

### Cytotoxicity Assay

Cytotoxicity assay was performed by using the MTT method as follows (19, 20). Different concentrations (50–400 μg/ml) of RCHP-S and mouse macrophages RAW 264.7 cells were treated for 48 h, after which the medium was discarded. A total of 0.5 mg/ml MTT was added to continue incubation in an incubator for another 4 h, after which the medium was discarded. The DMSO (100 μl) was added to dissolve blue-purple formazan in the cells. The absorbance was read at 570 nm with a microplate reader.

### NO-Release Inhibition Experiment

NO contributes to decreasing enzyme activity, protein, and mRNA levels of distinct P450 enzymes during inflammation (21). In a 96-well plate, macrophages RAW 264.7 were cultivated with a phenol red-free medium. After cells were grown to approximately 60%, different concentrations (50, 100, and 200 μg/ml) of RCHP-S were added to act on the cells for 1 h, after which 2 μg/ml of LPS was added. After 24 h, the cell culture medium supernatant was aspirated to generate a new 96-well plate, and then, Griess A and Griess B reagents were added to detect NO levels with a microplate reader at 540 nm. PBS was taken as a blank control.

### Anti-inflammatory Activity Test *in vitro*

To detect changes in inflammatory factor expression in mouse macrophages RAW 264.7 under different polysaccharide concentrations, real-time fluorescent quantitative polymerase chain reaction (PCR) was used to determine the level of gene transcription. RAW 264.7 cells were cultured overnight in a 6-well plate, then combined with different RCHP-S concentrations which were 50 μg/ml, 100 μg/ml, and 200 μg/ml, pre-incubated for 1 h, then combined with 2-μg/ml LPS for 24 h. Cells were then gently rinsed three times with preheated PBS, and total RNA was extracted from the cells according to the TRIzol instructions of the TransGen Company (Beijing, China).

The fold change in relative gene expression levels was calculated according to the 2^−Δ*ΔCt*^ equation. The RAW 264.7 cell anti-inflammatory level target gene-related primer sequence was as follows. The iNOS forward primer was 5′-ATGGAACAT CCCAAATACGA-3′, the reverse primer was 5′-GTCGTAGAGGACCAC TTT GT-3′, the TNF-α forward primer was 5′-CTTCTGTCTACTGAACTTCGGG-3′, the reverse primer was 5′-CAGGCTTGTCACTCGAATTTTG-3′, the IL-1β forward primer was 5′-ACG GACCCCAAAAGATGAAG-3′, the reverse primer was 5′-TTCTCCACAGCCACAATG AG-3′, the IL-6 forward primer was 5′-CAAAGCCAGAGTCCTTCAGAG-3′, and the reverse primer was 5′-GTCCTTAGCCACTCCTTCTG-3′. Reference gene for the GAPDH forward primer was 5′-GACATCAAGAAGGTGGTGAAGC-3′ and the reverse primer was 5′-GAAGGTGGA AGAGTGGGAGTT-3′.

Cytokine secretion was detected by ELISA. RAW264.7 cells (10^4^ cells/well) were seeded in 96-well plates, with a serum-free medium which was incubated overnight. Then, three concentrations of RCHP-S, which were 50 μg/ml, 100 μg/ml, and 200 μg/ml, were added and incubated for 12 h. Finally, the 2 μg/ml LPS was added to the model groups for another 2 h. The amount of IL-6 and TNF-α secretion in culture supernatants was determined by using an ELISA kit according to the manufacturer's instructions.

### Animal Experiment

According to previous literature reports (22, 23), we have made some modifications to the protocols. Kunming female 5-week-old mice were provided by the Henan University of Chinese Medicine, and animal experiments were approved by the Animal Ethics Committee of the Henan University of Chinese Medicine. Mice were housed under a controlled room temperature of 22°C and humidity of 55% on a 12-h light–dark cycle with free access to diets and water. After a week of adaptation, the experiment was divided into 6 mice per group, which were respectively gavaged according to RCHP-S low-dose group 50 mg/kg, high-dose group 200 mg/kg, and 50 mg/kg positive drug sulfasalazine, in addition to controlling mice with purified water once a day. This was then changed with a 4% dextran sulfate aqueous solution (DSS, 40 kDa, Aladdin, Shanghai) for 4–8 days to drink freely for 5 days. The mice showed different clinical symptoms, such as diarrhea and blood in the stool, indicating that the model was successfully established. DSS was removed at 9–13 days, and all mice were switched to drink freely purified water. The experimental group continued to be gavaged until sacrificed on the 14th day.

Enzyme-linked immunosorbent assay determination (24) was performed as follows: 100 mg colon tissue and 200 μl PBS were homogenized. The homogenate buffer was collected by centrifugation at 12,000 r/min for 10 min. The supernatant was taken to detect levels of IL-6 and TNF-α which were then measured according to the instructions and detected at 450 nm with a microplate reader.

Histological examination (25) was as follows. The colons of each mouse group were taken to measure their lengths and calculate statistics. Part of the colon from mice in each group was taken and fixed in 4% paraformaldehyde solution for 2 days, after which hematoxylin-eosin staining (HE) sections were performed.

The DAI score was calculated according to the following formula (26) (Supplementary Table S1). DAI = weight loss index + stool formation index + stool blood index. From experimental analysis on the first day, mice were weighed at a fixed time every day to observe the color and characteristics of the mouse stool.

### Statistical Analysis

All of the results are expressed as the mean ± SD. Statistical analysis was determined by using one-way ANOVA with GraphPad Prism 5.0 software. Statistical significance was chosen as ^*^p < 0.05, ^**^p < 0.01, and ^***^p < 0.001.

## Results and Discussion

### The Characteristic Structure of RCHP-S

#### Monosaccharide Composition

The crude polysaccharide of *R. chingii Hu* was isolated from *R. chingii* Hu, with a yield of 14.32%. Following the DEAE-52 column and gel chromatography column, a purified polysaccharide of *R. chingii Hu* was obtained named RCHP-S, and its yield was 3.46%.

**Figure 1:**
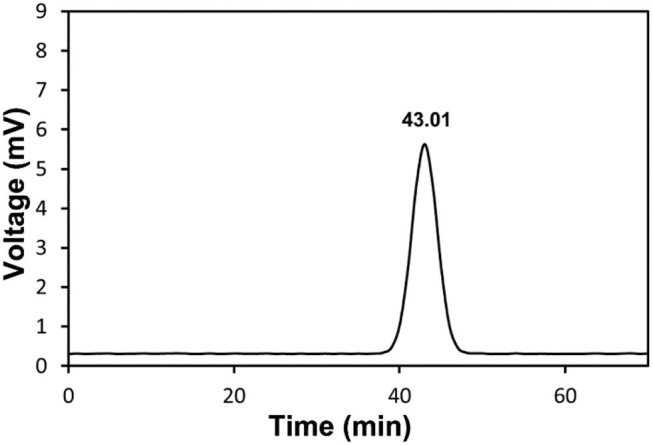
HPGPC chromatogram of RCHP-S.

As shown in Supplementary Table S2, the molecular weight of polysaccharides, the composition of monosaccharides, and the connection between monosaccharides and other structural characteristics determine polysaccharide type and their biological activity. Molecular weight and homogenization of RCHP-S were detected by HPGPC. There is a single and symmetrical peak in the HPGPC spectrum of RCHP-S, which showed that all RCHP-S were pure polysaccharides (Figure 1). The standard curve was log (Mw) = −0.1863T + 12.13 (*R*^2^ = 0.9876). According to the peak HPGPC time, the molecular weight of RCHP-S was calculated as 13.15 kDa. RCHP-S contains a lot of uronic acids, which was 48.32%, whereas RCHP-S protein content was <1%.

**Figure 2:**
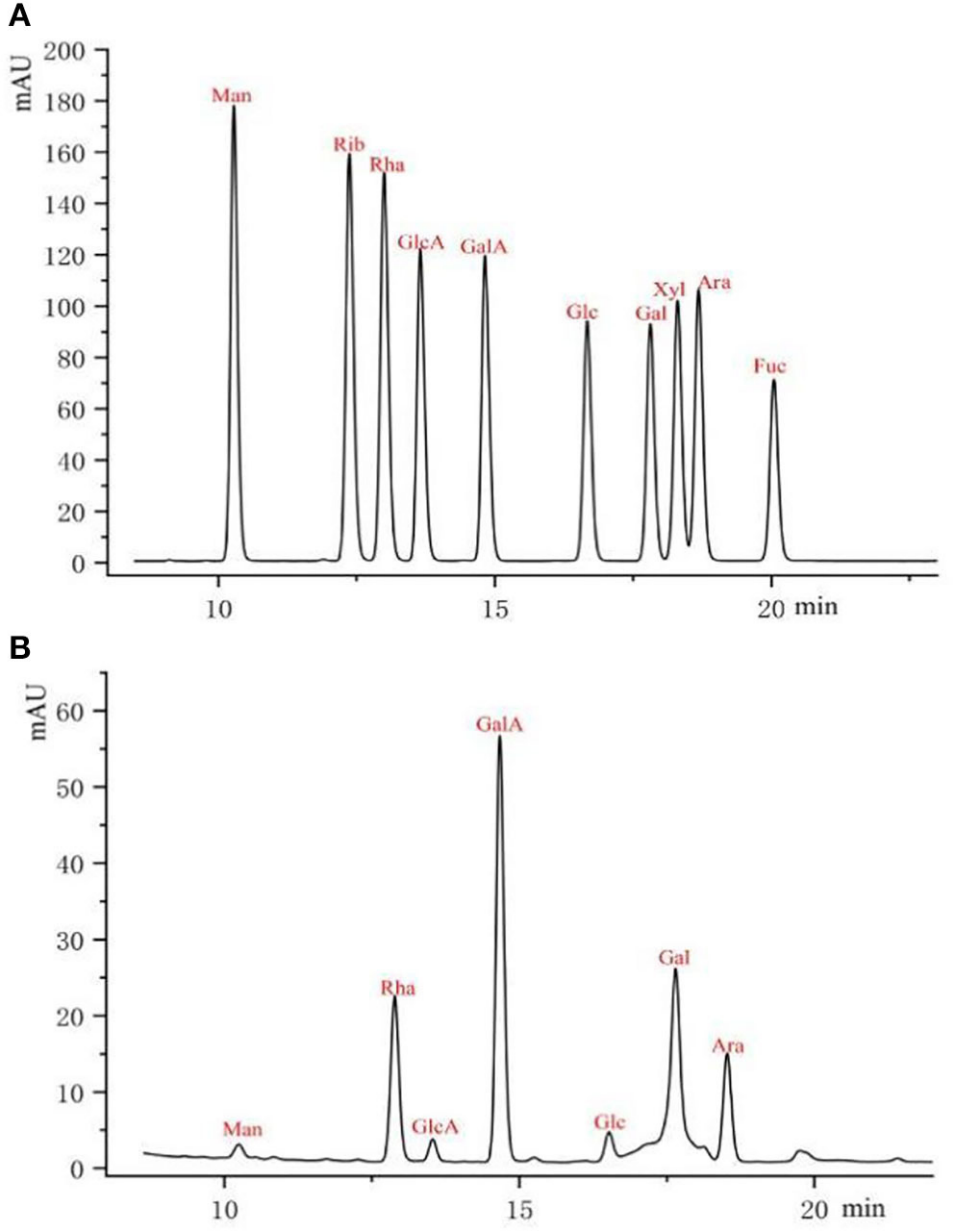
Pre-column PMP derivative HPLC of monosaccharide standards **(A)** and RCHP-S sample **(B)**.

As shown in Figure 2, pre-column PMP derivatization of RCHP-S was performed by HPLC to measure monosaccharide composition. The result shows that RCHP-S consisted of mannose, rhamnose, glucuronic acid, galacturonic acid, glucose, galactose, and arabinose with the molar ratio of 1.52:19.08:1.64:41.98:2.29:20.61:12.88 (Supplementary Table S2).

### Methylation Analysis

The five sugar residues were easy to describe as residues A–I in Table 1. Methylation analysis was an indispensable experimental method to study different glycosidic bonds in polysaccharides and their linkage methods. In this study, we performed methylation analysis on RCHP-S combined with nuclear magnetic resonance spectroscopy to jointly analyze the connection between monosaccharides in RCHP-S. As shown in Table 1 and Supplementary Figure S1, RCHP-S was mainly composed of nine derivatives, namely, 2,3,6-Me_3_-Gal, 2,3,4,6-Me_4_-Gal, 2,3-Me_2_-Gal, 3,4-Me_2_-Rha, 3-Me-Rha, 2,3-Me_2_-Ara, 2,3,5-Me_3_-Ara, 2,3,6-Me_3_-Glc, and 2,3,6-Me_3_-Man.

**Table 1:** Methylation analysis results of RCHP-S after reduction.

**Code**	**O-methylated alditol acetates**	**Linkage type**	**Ratio**	**Ions**
A	2,3,6-Me_3_-Gal	( → 4)-Gal*p*-(1 → )	3.8	59, 71, 87, 99, 102, 113, 118, 131, 142, 157, 173, 233
B	2,3,4,6-Me_4_-Gal	Glc*p*-(1 → )	0.8	59, 71, 87, 102, 118, 129, 145, 161, 162, 190, 205
C	2,3-Me_2_-Gal	( → 4,6)-Gal*p*-(1 → )	0.9	59, 85, 102, 118, 127, 142, 159, 187, 201, 261
D	3,4-Me_2_-Rha	( → 2)-Rha*p*-(1 → )	1.7	57, 71, 89, 100, 115, 130, 131, 190
E	3-Me-Rha	( → 2,4)-Rha*p*-(1 → )	1.1	59, 74, 88, 101, 130, 143, 190, 203
F	2,3-Me_2_-Ara	( → 5)-Ara*f*-(1 → )	0.8	59, 71, 87, 102, 118, 129, 189
G	2,3,5-Me_3_-Ara	Ara*f*-(1 → )	0.8	59, 71, 87, 102, 118, 129, 145, 162
H	2,3,6-Me_3_-Glc	( → 4)-Glc*p*-(1 → )	–	59, 71, 87, 99, 102, 113, 118, 129, 131, 142, 159, 233
I	2,3,6-Me_3_-Man	( → 4)-Man*p*-(1 → )	–	59, 71, 87, 99, 102, 113, 118, 129, 143, 162, 233

### NMR Analysis

The chemical shifts of carbon and hydrogen on RCHP-S were assigned by HSQC and ^1^H-^1^H COSY. The results are summarized in Supplementary Table S3.

**Figure 3:**
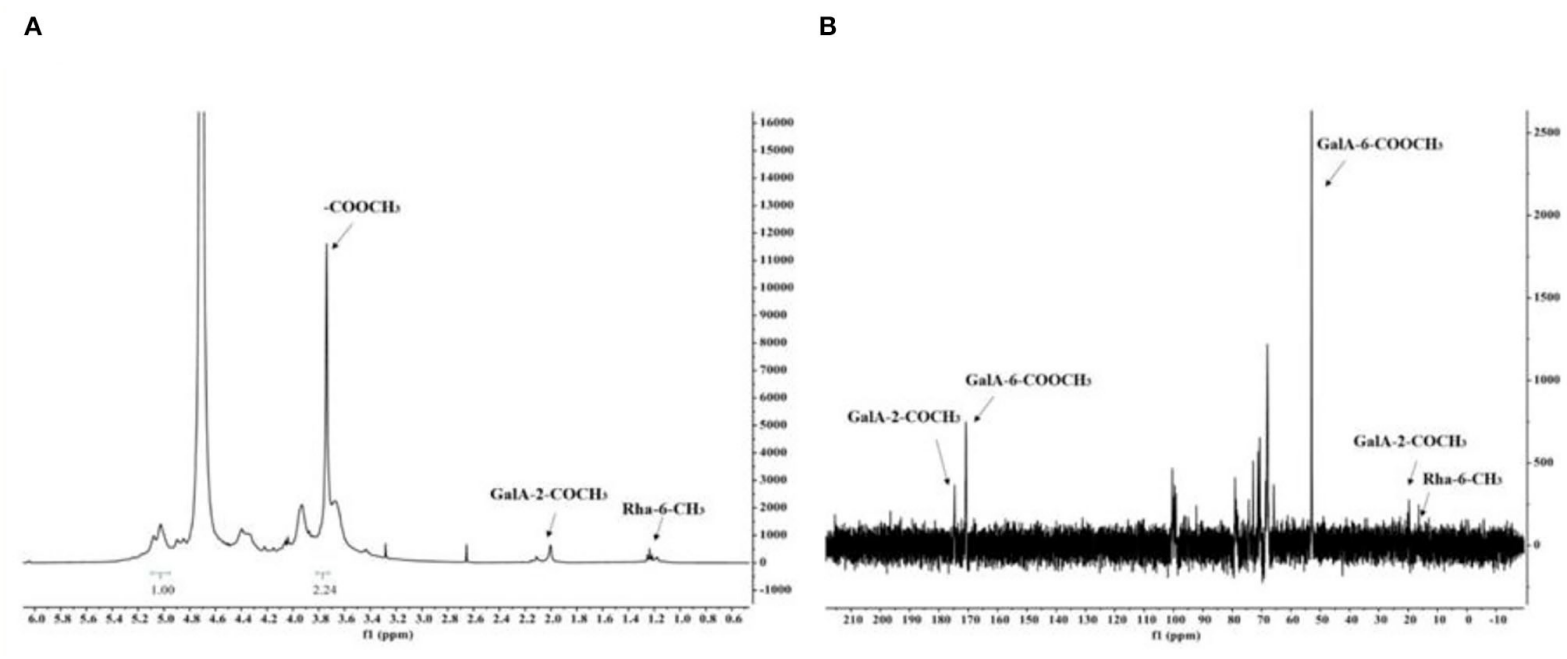
The ^1^H NMR spectra **(A)** and ^13^C NMR spectra **(B)** of RCHP-S.

There are strong signals at position δ3.78 in the ^1^H NMR spectrum of RCHP-S (Figure 3A), indicating that the sugar residue GalA was methylated. According to the ratio of the integral hydrogen spectrum of methyl ester and the integral ratio of anomeric carbon points, the proportion of sugar residue GalA methyl ester was ~74.67%. The signal at δ1.98 indicates the 2-position hydroxyl group part of the sugar residue GalA was acetylated. The signal at δ1.25 represents the methyl group at position 6 of the sugar residue Rha.

In the ^13^C NMR spectrum of RCHP-S shown in Figure 3B, the signal appeared in the sugar anomeric carbon region combined with the result of methylation analysis to give a signal of 100.4 ppm for C-1 of residue A. In addition, 170.8 ppm and 175.2 ppm were signals for C-6 and acetyl groups of residue A, which also verified that most 6-position carboxyl groups of GalA in RCHP-S were methylated and part of the 2-position hydroxyl groups were acetylated. C-6 signals of residues D and E appeared in the 16.5 ppm region, which is the typical methyl signal peak for rhamnose.

**Figure 4:**
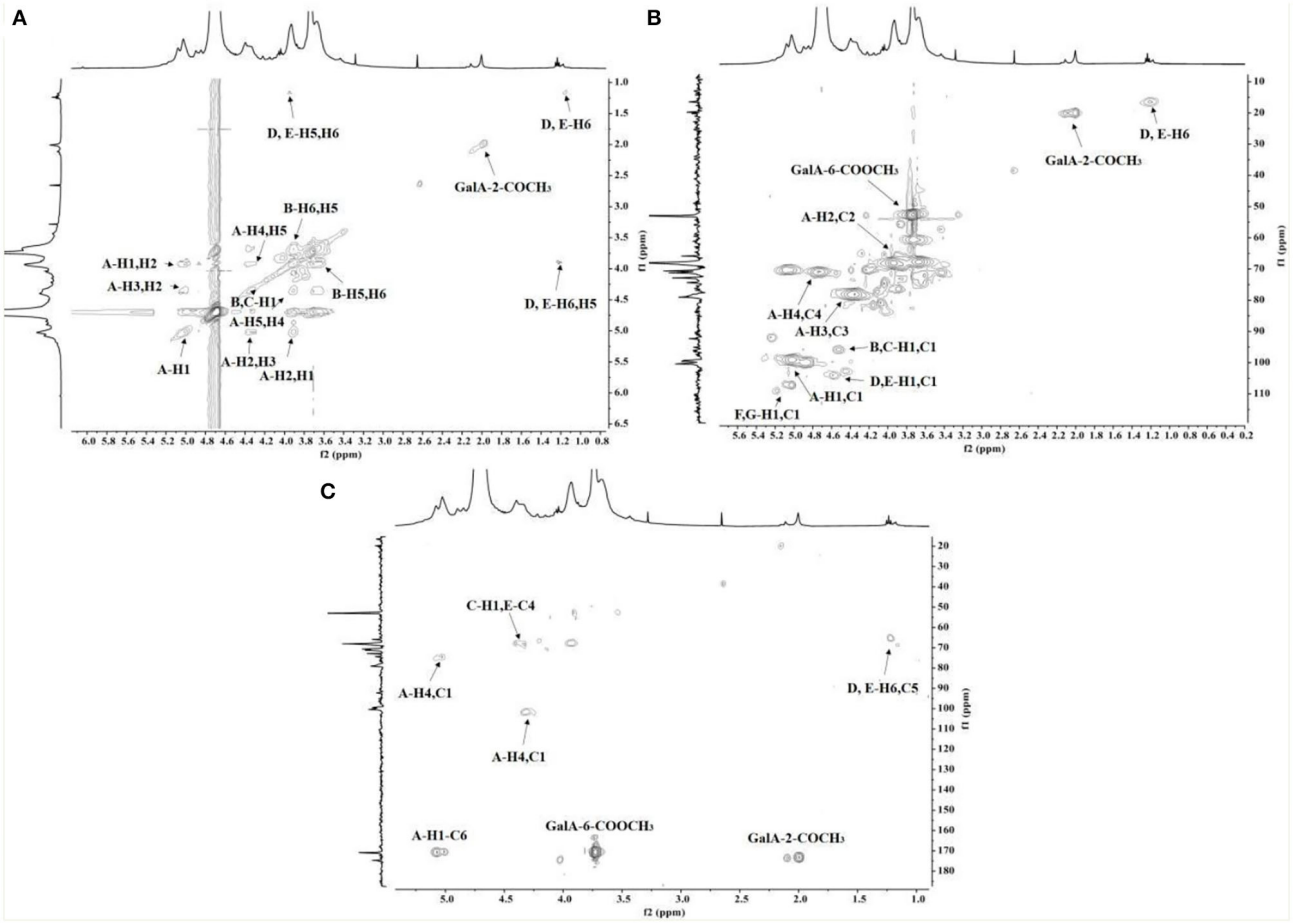
The **(A)** COSY, **(B)** HSQC, and **(C)** HMBC spectra of RCHP-S.

**Figure 5:**
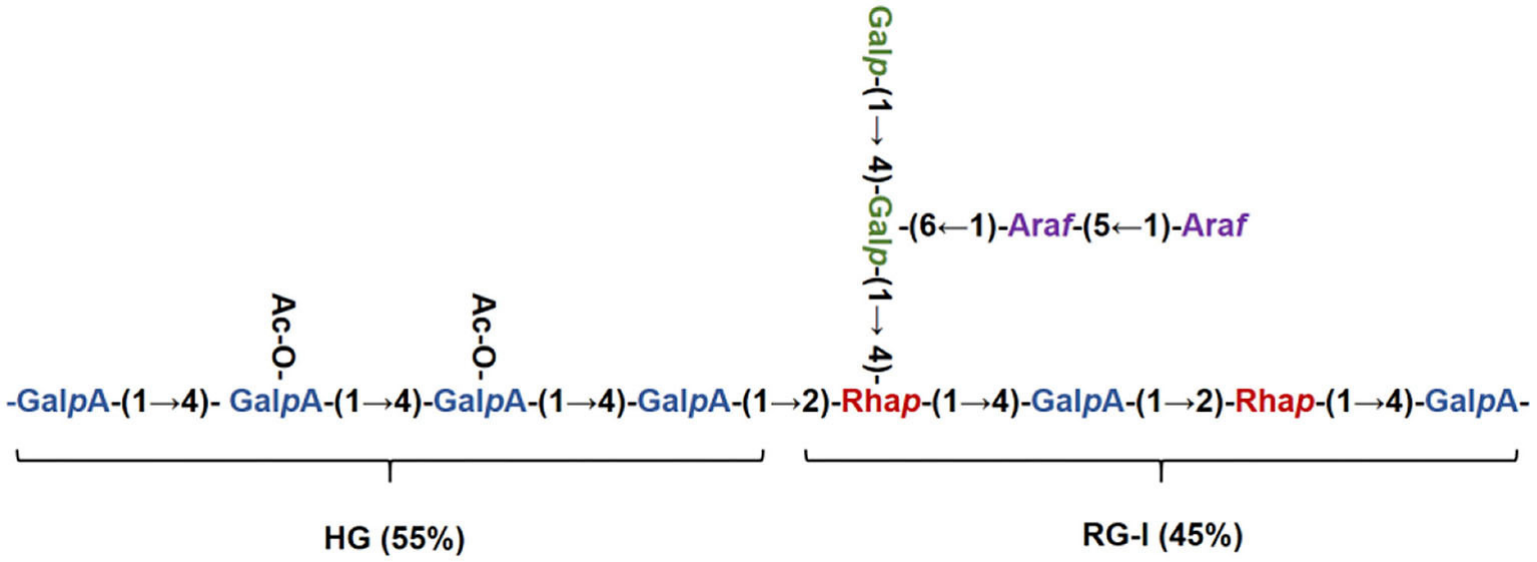
Structure schematic diagram of RCHP-S.

The chemical shifts of carbon and hydrogen for RCHP-S were assigned by ^1^H-^1^H COSY (Figure 4A) and HSQC (Figure 4B). In the HMBC (Figure 4C) spectrogram, the significant cross-peaks of (A-C4, A-H1) and (A-C1 and A-H4) are shown, indicating that most of the residue A was self-phased by the O-4 link. The appearance of cross-peaks (C-H1 and E-C4) indicates residue C is linked to residue E *via* O-4. Results show RCHP-S was mixed pectin composed of HG-type and RG-I (Figure 5). Combined with the results of the methylation analysis, we could draw the following conclusions. Polysaccharide RCHP-S was a highly methyl esterified pectin. The main chain of polysaccharide RCHP-S was composed of 1,4-Gal A and 1,2-Rha. Furthermore, this pectin contains two structural regions, namely, the HG type of the smooth zone and the RG1 type of the rough zone. Based on the above information, the inferred structure of the polysaccharide is shown in Figure 5.

### Effects of RCHP-S on the Survival Rate of Mouse Macrophages RAW264.7

In recent years, pectin polysaccharides had been an area of interest for many researchers, as they possess many activities, such as good biocompatibility with human cells, low toxic side effects, and so on. As shown in Supplementary Figure S2, RCHP-S has no obvious cytotoxic effect on RAW 264.7 cells when the concentration range is between 50 and 400 μg/ml, which indicates that the polysaccharide RCHP-S has good biocompatibility.

### Effects of RCHP-S on NO Production and Phagocytosis

The functions of endothelial cells (such as vascular endothelial cell) will be changed in inflammation, with nitric oxide production changing, pro-inflammatory chemokines and cytokines increasing, resulting in the recruitment of immune cells into inflamed sites (27). Nitric oxide (NO) plays the role of an inflammatory indicator where it would accumulate as the hepatocytes injuries (28). When compared with the blank control, the 2 μg/ml LPS in the positive control group significantly increased NO secretion from RAW264.7 cells after 24 h. Cells were pre-treated with polysaccharides for 1 h and then incubated with LPS. For high concentration (200 μg/mL) *p* < 0.01, medium concentration (100 μg/mL) *p* < 0.01 and low concentration (50 μg/mL) *p* < 0.05, different dose groups could reduce cellular NO secretion (Supplementary Figure S3).

### Effect of Different RCHP-S Concentrations on Cytokine Secretion

**Figure 6:**
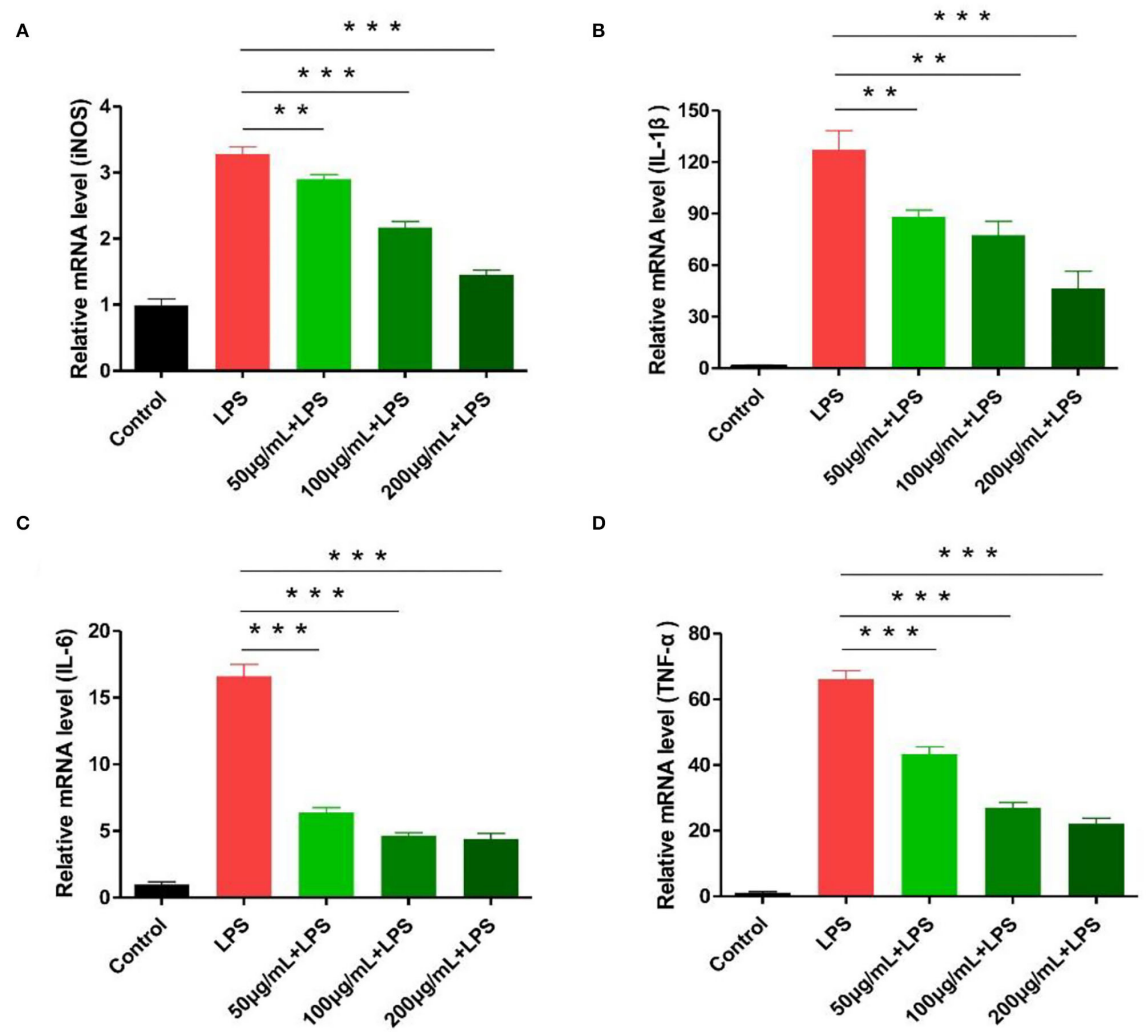
Effect of different RCHP-S concentrations on cytokine secretion [**(A)** iNOS, **(B)** IL-1β, **(C)** IL-6, and **(D)** TNF-α; **p < 0.01, and ***p < 0.001].

Experimental results showed nitric oxide synthase iNOS and common inflammatory factors (IL-1β, IL-6, TNF-α) play a key role in inflammation development (29, 30). Following LPS-stimulated macrophages, the expression of iNOS, IL-1β, IL-6, and TNF-α related genes increased significantly. In the polysaccharide experimental group, cells were pre-treated with different polysaccharide concentrations, showing a significant inhibitory effect on iNOS and IL-1β genes (*p* < 0.05). Similarly, IL-6 and TNF-α expression showed more effective results (*p* < 0.01; Figure 6). We calculated the expression of IL-6 and TNF-α detected by ELISA in RAW264.7 cells. The expression of IL-6 and TNF-α was significantly inhibited after RCHP-S was added. The results of cytokine expression level and RT-PCR were consistent (Supplementary Figure S4).

### Animal Experiments

**Figure 7:**
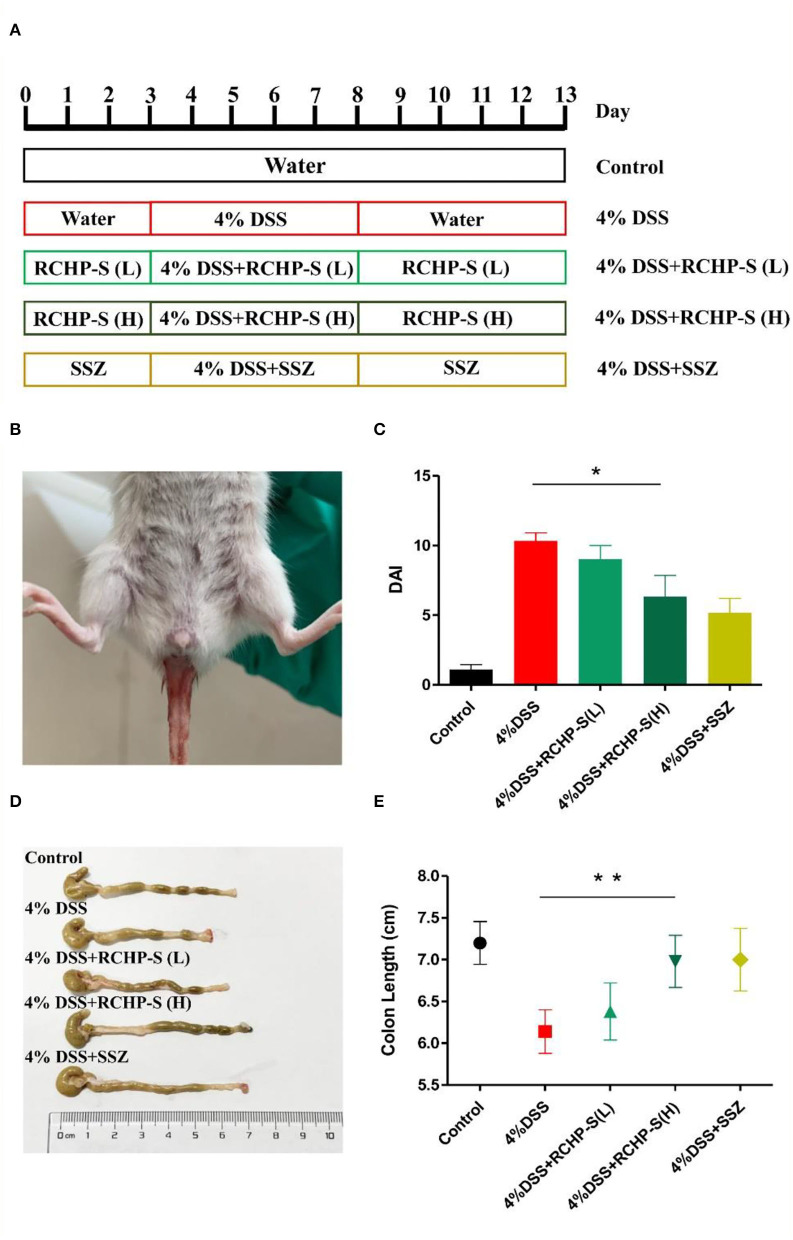
**(A)** Establishment model of mouse enteritis, **(B)** clinical symptoms of colitis (hematochezia) in mice, **(C)** DAI scores, **(D)** colon morphology from mice in different groups, and **(E)** change in colon length. *p < 0.05, **p < 0.01.

As shown in Figure 7A, the mice received intragastric administration according to the procedure described. Blank control mice had normal diets and drinking water, with no significant weight loss or blood in the stool. For the DSS group, after drinking DSS, mice showed obvious clinical symptoms after the third day (Figure 7B). The main manifestations were rough coat color, weight loss, loose stools, and bloody stools, but no deaths. Through the administration of polysaccharides and anti-inflammatory drugs, we found scores for the high-dose polysaccharide and DSS groups were significantly different (*p* < 0.05). DAI score results are shown in Figure 7C, indicating that the high-dose polysaccharide group can effectively alleviate colitis symptoms and protect the colon.

Based on anatomy, the DSS-induced mouse colon was significantly shorter than the normal group (Figure 7D). For the RCHP-S high-dose group, the mouse colon length (*n* = 6) was significantly different from the model group (*p* < 0.01; Figure 7E), indicating that RCHP-S could effectively reduce colon inflammation and be used as a natural anti-inflammatory drug.

**Figure 8:**
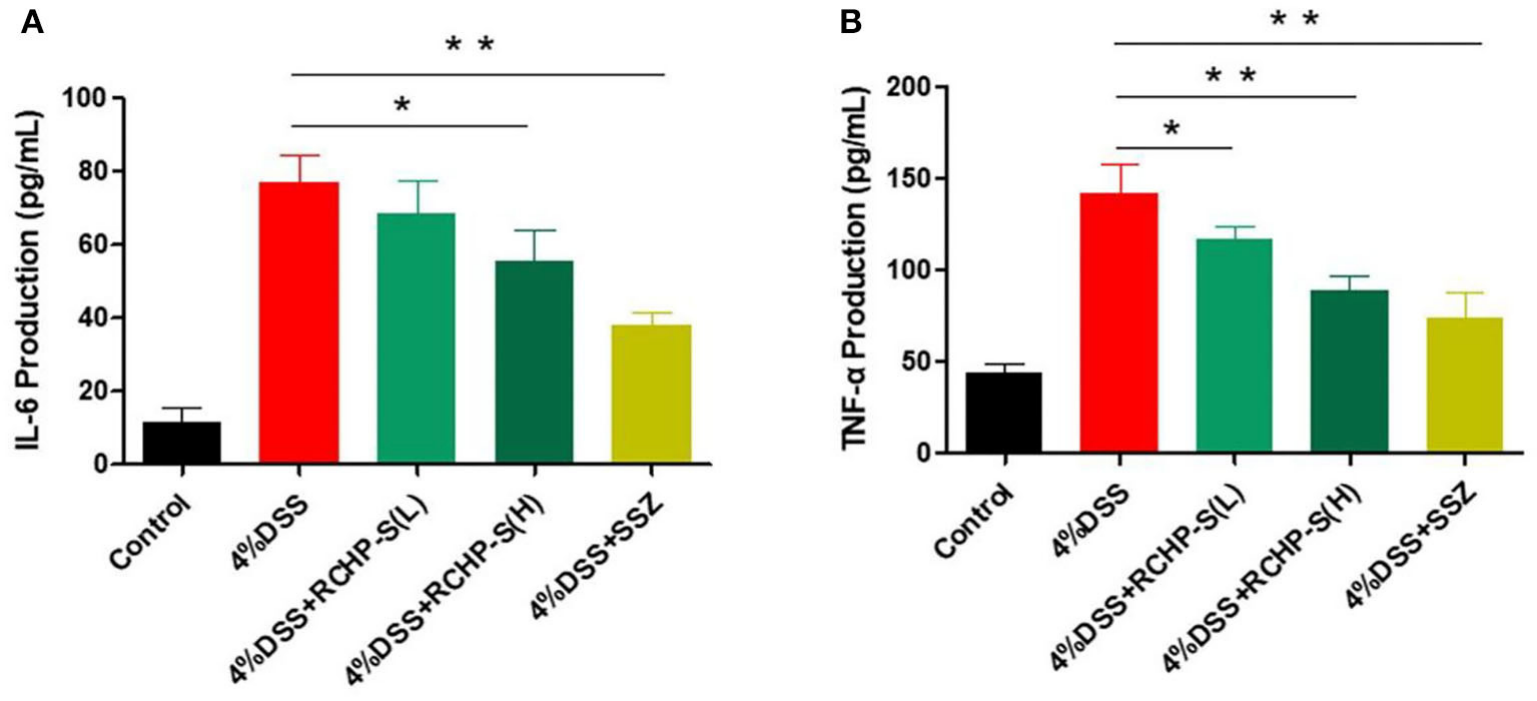
ELISA measurements of **(A)** IL-6 and **(B)** TNF-α levels in colon tissue. *p < 0.05, **p < 0.01.

Inflammatory factors play a key role in inducing enteritis. We detected IL-6 and TNF-α in colon tissue and found that the IL-6 and TNF-α content in the model group was significantly higher than in the blank control group (Figure 8). In comparison to the DSS group, the high dose of the polysaccharide group is significantly reduced. In the colon tissue, IL-6 plays a very important role in regulating inflammatory bowel disease. Chand et al. reported that STAT3 signaling *via* the IL-6ST/gp130 cytokine receptor promotes epithelial integrity and intestinal barrier function during DSS-induced colitis (31).

Ulcerative colitis (UC) is a chronic nonspecific inflammatory disease, which exhibited abdominal pain, diarrhea, and so on (32). Ulcerative colitis may be that the two conditions result from an inappropriate and exaggerated mucosal immune response to constituents of the intestinal flora in a genetically susceptible host (33). But there is no definitive cure for UC (34), except for steroids and non-steroidal anti-inflammatory drugs, which have serious side effects with long-term use, including hyperlipidemia, systemic infection, and herpes zoster infection (35).

Pectin, majorly comprised of galacturonic acid, is a non-cellulosic polysaccharide, which is intricately connected with the cellulose and hemicellulose units (36) but had some differences in composition, structure, and molecular weight (37). The pectin that has a high number of neutral sugar side chains ameliorates colonic tissue damage and decreases colonic IL-1β and IL-6 levels to attenuate DSS- and 2,4,6-TNBS-induced experimental colitis. In addition, the protective effect of pectin against experimental colitis is mediated in a side chain-dependent manner (38). Many studies show that Tremella polysaccharide has a molecular weight of 5.17 × 10^4^ Da, which is mainly composed of mannose, glucose, and a small amount of galactose, glucuronic acid, xylose, and arabinose. It can effectively improve the clinical symptoms of colitis. The length of the colon is 13.85% longer than that of the model group, and the spleen index is 11.95% lower than that of the model group (39).

Our results show that RCHP-S can effectively inhibit cytokines, thereby improving enteritis symptoms. Detection of tumor necrosis factor TNF-α showed that the low-dose group of RCHP-S can inhibit its secretion (Figure 8B). The results indicate that polysaccharides can improve intestinal inflammation by inhibiting inflammatory factors, which were similar to cell experiments.

**Figure 9:**
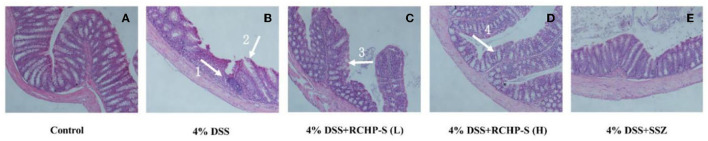
Haematoxylin-eosin (HE) staining of colon among the different groups (200 ×). **(A)** Control. **(B)** 4% DSS. **(C)** 4% DSS+RCHP-S (L). **(D)** 4% DSS+RCHP-S (H). **(E)** 4% DSS+SSZ (arrow 1 indicates the accumulation of inflammatory cells; arrow 2 and 3 indicate the loss of crypts; arrow 4 indicates the intact crypts).

Using HE staining and colonic pathology observations, we found that mucosal damage, basal inflammatory cell infiltration, abnormal crypt structure, and epithelial villi defect occurred in the DSS group compared to the negative control group. In the low-dose RCHP-S group, some crypts were missing (arrow 3), but in the high-dose group, the colonic mucosa was basically intact, and epithelium and crypt morphology (arrow 4) remained essentially unchanged (Figure 9).

In summary, we evaluated the anti-inflammatory effects of RCHP-S using cell and animal experiments. The colitis model was established, and RCHP-S was used for treatment. This study showed that RCHP-S can reduce DSS-induced enteritis to produce inflammatory factors and show RCHP-S can alleviate inflammatory responses of DSS in the intestinal tract.

## Conclusion

A novel pectin polysaccharide RCHP-S was extracted and purified from *R. chingii* Hu. The RCHP-S was consisted of mannose, rhamnose, glucuronic acid, galacturonic acid, glucose, galactose, and arabinose in a molar ratio of 1.52:19.08:1.64:41.98:2.29:20.61:12.88 and was mainly composed of HG-type pectin domains, and also contains a small amount of RG-I. The HG domain in RCHP-S had a high degree of methyl esterification, and part of the 2-position hydroxyl group was modified by acetylation. The RAW 264.7 cells were pretreated with RCHP-S could show a significant inhibitory effect on the mRNA level of iNOS, IL-1β, IL-6, and TNF-α *in vitro*. These findings suggest that polysaccharides from *R. chingii* Hu show good anti-inflammatory activity and DSS-induced colitis can be improved for anti-inflammatory agent development.

## Data Availability Statement

The original contributions presented in the study are included in the article/Supplementary Material, further inquiries can be directed to the corresponding author/s.

## Ethics Statement

The animal study was reviewed and approved by Animal Ethics Committee of Henan University of Chinese Medicine.

## Author Contributions

YK performed research and carried out the data analyses. YH and JL produced the initial draft of the manuscript. JC contributed to the revision of the manuscript. YQ and CD designed the study. All authors contributed to the article and approved the submitted version.

## Funding

This work was financially supported by the Henan Provincial Science and Technology Research Project (222102310231 and 222102310624), Zhongjing Scholars Research Funding of Henan University of Chinese Medicine (00104311-2021-1-8), China Postdoctoral Science Fund Project (2021M690936), and MiaoPu Research Funding of Henan University of Chinese Medicine (MP2021-27 and MP2021-15).

## Conflict of Interest

The authors declare that the research was conducted in the absence of any commercial or financial relationships that could be construed as a potential conflict of interest.

## Publisher's Note

All claims expressed in this article are solely those of the authors and do not necessarily represent those of their affiliated organizations, or those of the publisher, the editors and the reviewers. Any product that may be evaluated in this article, or claim that may be made by its manufacturer, is not guaranteed or endorsed by the publisher.

## References

[1] Yan Y Ren F Wang P Sun Y Xing J. Synthesis and evaluation of a prodrug of 5-aminosalicylic acid for the treatment of ulcerative colitis. Iran J Basic Med Sci. (2019) 22:1452–61. 10.22038/IJBMS.2019.13991 32133064 PMC7043877

[2] Niu W Chen X Xu R Dong H Yang F Wang Y. Polysaccharides from natural resources exhibit great potential in the treatment of ulcerative colitis: a review. Carbohydr Polym. (2021) 254:117189. 10.1016/j.carbpol.2020.117189 33357839

[3] Wang Y Liu XP Zhao ZB Chen JH Yu CG. Expression of CD4+ forkhead box P3 (FOXP3)+ regulatory T cells in inflammatory bowel disease. J Dig Dis. (2011) 12:286–94. 10.1111/j.1751-2980.2011.00505.x 21791023

[4] Liao N Chen S Ye X Ye X Liu D. Structural characterization of a novel glucan from Achatina fulica and its antioxidant activity. J Agric Food Chem. (2014) 62:2344–52. 10.1021/jf403896c 24383933

[5] Xie Z Wang Y Huang J Qian N Shen G. Anti-inflammatory activity of polysaccharides from Phellinus linteus by regulating the NF-κB translocation in LPS-stimulated RAW2647 macrophages. Int J Biol Macromol. (2019) 129:61–7. 10.1016/j.ijbiomac.2019.02.023 30731160

[6] Hou C Chen L Yang L Ji X. An insight into anti-inflammatory effects of natural polysaccharides. Int J Biol Macromol. (2020) 153:248–55. 10.1016/j.ijbiomac.2020.02.315 32114173

[7] Bai Y Jia X Huang F Zhang R Zhang M. Structural elucidation, anti-inflammatory activity and intestinal barrier protection of longan pulp polysaccharide LPIIa. Carbohydr Polym. (2020) 246:116532. 10.1016/j.carbpol.2020.116532 32747231

[8] Meng Y Xu Y Chang C Qiu Z Hu J Wu Y. Extraction, characterization and anti-inflammatory activities of an inulin-type fructan from *Codonopsis pilosula*. Int J Biol Macromol. (2020) 163:1677–86. 10.1016/j.ijbiomac.2020.09.117 32979437

[9] Chen L Xin X Zhang H Yuan Q. Phytochemical properties and antioxidant capacities of commercial raspberry varieties. J Funct Foods. (2013) 5:508–15. 10.1016/j.jff.2012.10.009

[10] Zhang TT Wang M Yang L Jiang JG Zhao JW Zhu W. Flavonoid glycosides from Rubus chingii Hu fruits display anti-inflammatory activity through suppressing MAPKs activation in macrophages. J Funct Foods. (2015) 18:235–43. 10.1016/j.jff.2015.07.006

[11] Zhang TT Lu CL Jiang JG Wang M Wang DM Wei Z. Bioactivities and extraction optimization of crude polysaccharides from the fruits and leaves of *Rubus chingii* Hu. Carbohydr Polym. (2015) 130:307–15. 10.1016/j.carbpol.2015.05.012 26076631

[12] Ke H Bao T Chen W. New function of polysaccharide from *Rubus chingii* Hu: protective effect against ethyl carbamate induced cytotoxicity. J Sci Food Agric. (2021) 101:3156–64. 10.1002/jsfa.10944 33211321

[13] Sun Y Shi X Zheng X Nie S Xu X. Inhibition of dextran sodium sulfate-induced colitis in mice by baker's yeast polysaccharides. Carbohydr Polym. (2019) 207:371–81. 10.1016/j.carbpol.2018.11.087 30600019

[14] Masuko T Minami A Iwasaki N Majima T Nishimura S Lee YC. Carbohydrate analysis by a phenol-sulfuric acid method in microplate format. Anal Biochem. (2005) 339:69–72. 10.1016/j.ab.2004.12.001 15766712

[15] Blumenkranz B. New method for quantitative determination of uronic acid. Anal Biochem. (1973) 54:484–9. 10.1016/0003-2697(73)90377-1 4269305

[16] Liu Y Cao S Li K Li SH Song CW Qin C. Rapid protein quantitation in pre two-dimensional electrophoresis by near-infrared imaging. J Anal Sci. (2017) 33:541–4. 10.13526/j.issn.1006-6144.2017.04.020

[17] Honda S Akao E Suzuki S Okuda M Kakehi K Nakamura J. High-performance liquid chromatography of reducing carbohydrates as strongly ultraviolet-absorbing and electrochemically sensitive 1-phenyl-3-methyl-5-pyrazolone derivatives. Anal Biochem. (1989) 180:351–7. 10.1016/0003-2697(89)90444-2 2817366

[18] Zhang P Sun F Cheng X Li X Mu H Wang S. Preparation and biological activities of an extracellular polysaccharide from *Rhodopseudomonas palustris*. Int J Biol Macromol. (2019) 131:933–40. 10.1016/j.ijbiomac.2019.03.139 30905754

[19] Ye H Wang K Zhou C Liu J Zeng X. Purification, antitumor and antioxidant activities in vitro of polysaccharides from the brown seaweed *Sargassum pallidum*. Food Chem. (2008) 111:428–32. 10.1016/j.foodchem.2008.04.012 26047446

[20] Wu X Mao G Zhao T Zhao J Li F Liang L. Isolation, purification and in vitro anti-tumor activity of polysaccharide from *Ginkgo biloba* sarcotesta. Carbohydr Polym. (2011) 86:1073–6. 10.1016/j.carbpol.2011.04.069

[21] Morgan ET Skubic C Lee CM Cokan KB Rozman D. Regulation of cytochrome P450 enzyme activity and expression by nitric oxide in the context of inflammatory disease. Drug Metab Rev. (2020) 52:455–71. 10.1080/03602532.2020.1817061 32898444 PMC7709541

[22] Sayer B Lu J Green C Söderholm JD Akhtar M McKay DM. Dextran sodium sulphate-induced colitis perturbs muscarinic cholinergic control of colonic epithelial ion transport. Br J Pharmacol. (2002) 135:1794–800. 10.1038/sj.bjp.0704633 11934821 PMC1573298

[23] Sina C Arlt A Gavrilova O Midtling E Kruse ML Müerköster SS. Ablation of gly96/immediate early gene-X1 (gly96/iex-1) aggravates DSS-induced colitis in mice: role for gly96/iex-1 in the regulation of NF-κB. Inflamm Bowel Dis. (2010) 16:320–31. 10.1002/ibd.21066 19714745 PMC3927407

[24] Wang J Luo X Cai S Sun J Wang S Wei X. Blocking HOTAIR protects human chondrocytes against IL-1β-induced cell apoptosis, ECM degradation, inflammatory response and oxidative stress via regulating miR-222-3p/ADAM10 axis. Int Immunopharmacol. (2021) 98:107903. 10.1016/j.intimp.2021.107903 34192661

[25] Gao H Zhao Q Song Z Yang Z Wu Y Tang S. PGLP-1, a novel long-acting dual-function GLP-1 analog, ameliorates streptozotocin-induced hyperglycemia and inhibits body weight loss. FASEB J. (2017) 31:3527–39. 10.1096/fj.201700002R 28461341

[26] Hu LH Fan YJ Li Q Guan JM Qu B Pei FH. Bortezomib protects against dextran sulfate sodium-induced ulcerative colitis in mice. Mol Med Rep. (2017) 15:4093–9. 10.3892/mmr.2017.6524 28487944 PMC5436237

[27] Hao J Shen SM Jie Y Zhang PP Shi Y Balaji M. Sphingosine 1-phosphate receptor 1 (S1PR1) agonist CYM5442 inhibits expression of intracellular adhesion molecule 1 (ICAM1) in endothelial cells infected with influenza A viruses. PLoS ONE. (2017) 12:e0175188. 10.1371/journal.pone.0175188 28399143 PMC5388330

[28] Li J. Determinants of nitric oxide protection and toxicity in liver. Am J Physiol. (1999) 276:G1069–73. 10.1152/ajpgi.1999.276.5.G1069 10329995

[29] Gholamrezayi A Mohamadinarab M Rahbarinejad P Fallah S Tavakoli T. Characterization of the serum levels of Meteorin-like in patients with inflammatory bowel disease and its association with inflammatory cytokines. Lipids Health Dis. (2020) 19:230. 10.1186/s12944-020-01404-6 33126870 PMC7602304

[30] Karmacharya U Regmi SC Awasthi BP Chaudhary P Kim YE Lee IH. Synthesis and activity of N -(5-hydroxy-3,4,6-trimethylpyridin-2-yl)acetamide analogues as anticolitis agents via dual inhibition of TNF-α- and IL-6-induced cell adhesions. Bioorg Med Chem Lett. (2021) 43:128059. 10.1016/j.bmcl.2021.128059 33895277

[31] Pang L Huynh J Alorro MG Li X Chand AL. STAT3 Signalling via the IL-6ST/gp130 cytokine receptor promotes epithelial integrity and intestinal barrier function during DSS-induced colitis. Biomedicines. (2021) 9:187. 10.3390/biomedicines9020187 33673239 PMC7918037

[32] Wang ZG Ying XG Gao P Wang CL Luo HY. Anti-inflammatory activity of a peptide from skipjack (*Katsuwonus pelamis*). Mar Drugs. (2019) 17:582. 10.3390/md17100582 31614893 PMC6835902

[33] Latiano A Palmieri O Latiano T Corritore G Bossa F Martino G. Investigation of multiple susceptibility loci for inflammatory bowel disease in an Italian Cohort of patients. PLoS ONE. (2011) 6:e22688. 10.1371/journal.pone.0022688 21818367 PMC3144927

[34] Bartoszek A Makaro A Bartoszek A Fichna J Salaga M. Walnut oil alleviates intestinal inflammation and restores intestinal barrier function in mice. Nutrients. (2020) 12:1302. 10.3390/nu12051302 32370215 PMC7284466

[35] Sandborn WJ Su C Sands BE D'Haens GR Vermeire S Schreiber S. Tofacitinib as induction and maintenance therapy for ulcerative colitis. New Engl J Med. (2017) 376:1723–36. 10.1056/NEJMoa1606910 28467869

[36] Kameshwar AKS Qin W. Comparative study of genome-wide plant biomass-degrading CAZymes in white rot, brown rot and soft rot fungi. Mycology. (2017) 9:93–105. 10.1080/21501203.2017.1419296 30123665 PMC6059041

[37] Al-Asmar A Giosafatto CVL Sabbah M Mariniello L. Hydrocolloid-based coatings with nanoparticles and transglutaminase crosslinker as innovative strategy to produce healthier fried kobbah. Foods. (2020) 9:698. 10.3390/foods9060698 32492773 PMC7353631

[38] Ishisono K Mano T Yabe T Kitaguchi K. Dietary Fiber Pectin ameliorates experimental colitis in a neutral sugar side chain-dependent manner. Front Immunol. (2019) 10:2979. 10.3389/fimmu.2019.02979 31921214 PMC6930924

[39] Xu Y Xie L Zhang Z Zhang W Peng W. Tremella fuciformis polysaccharides inhibited colonic inflammation in dextran sulfate sodium-treated mice via Foxp3+ T cells, gut microbiota, and bacterial metabolites. Front Immunol. (2021) 12:648162. 10.3389/fimmu.2021.648162 33868283 PMC8049506

