# Supplementary material for: Anti-inflammatory Effect of a Novel Pectin Polysaccharide From Rubus chingii Hu on Colitis Mice
Source: Front Nutr. 2022 Apr 29;9:868657. doi: 10.3389/fnut.2022.868657 (PMC9105459; doi:10.3389/fnut.2022.868657)
Supplement: Supplementary file 1 [file Data_Sheet_1.docx]

Supplementary Material

# Supplementary Figures and Tables

## Supplementary Figures


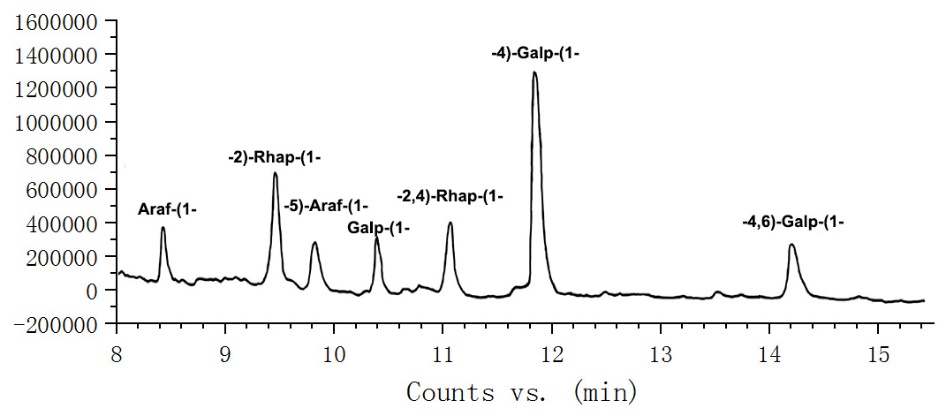


**Fig. S1.** GC-MS total ion current chromatogram of PMAAs for RCHP-S.


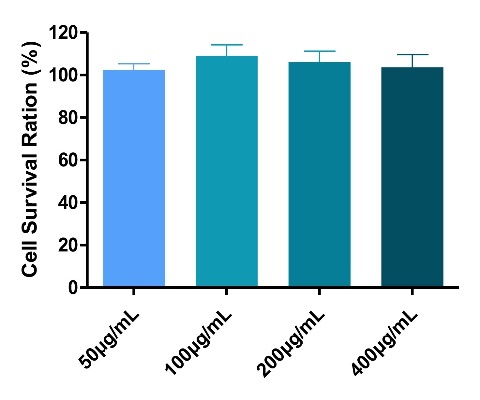


**Fig. S2.** Effect of different RCHP-S concentrations on survival rate of RAW264.7 cells.


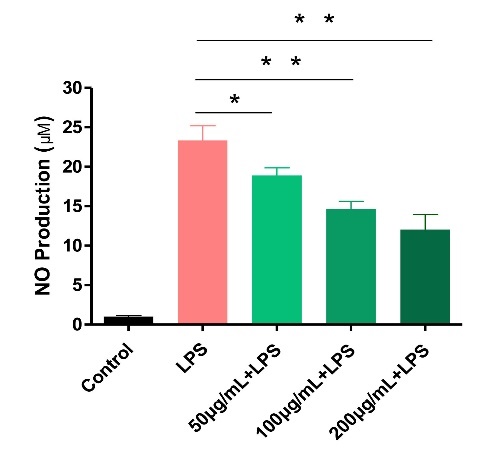


**Fig. S3.** Effect of different RCHP-S concentrations on NO secretion in mouse macrophages.

**
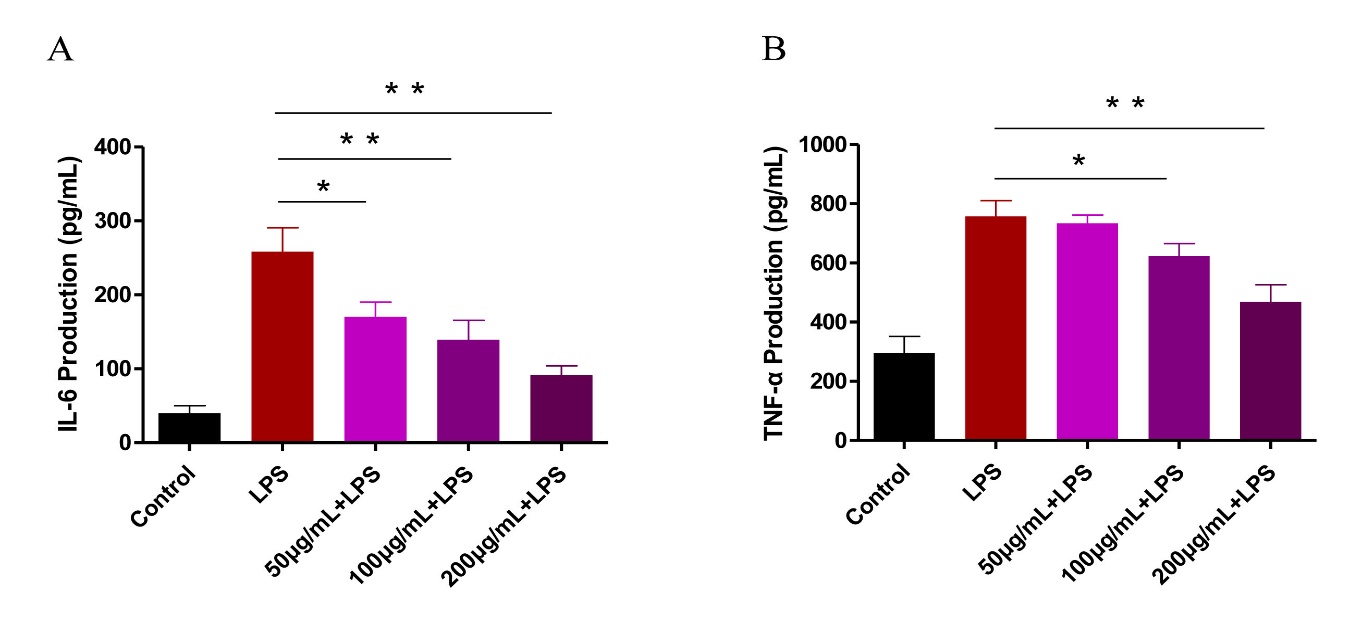
**

**Fig. S4.** The ELISA measurements of (A) IL-6 and (B) TNF-α levels in RAW264.7 cells.

## Supplementary Tables

**Table S1.** DAI scoring criteria

| Degree of weight loss (%) | Stool traits | Occult blood (blood stool) | Scoring |
| --- | --- | --- | --- |
| 0 | Normal | Normal | 0 |
| 1~5 |  |  | 1 |
| 5~10 | Loose | Occult blood positive | 2 |
| 10~15 |  |  | 3 |
| >15 | Loose stool | Gross bloody stool | 4 |

Note: Normal stool: soft and hard shaped

Loose stool: stool is not formed but does not adhere to the mouse anus

Loose stool: Stool is not shaped and can adhere to the mouse's anus

**Table S2.** Characteristic structure of RCHP-S

| **Item** | **GBLPW** |
| --- | --- |
| Carbohydrate (%) | 57.13±2.43 |
| Protein (%) | 0.53±0.03 |
| Uronic acid (%) | 48.32±3.23 |
| Molecular (kDa) | 13.15 |
| **Monosaccharide composition (%)** |  |
| Man | 1.52 |
| Rha | 19.08 |
| Glc A | 1.64 |
| GalA | 41.98 |
| Glc | 2.29 |
| Gal | 20.61 |
| Ara | 12.88 |

**Table S3.** Chemical shift assignments of RCHP-S

| Code | H1 | H2 | H3 | H4 | H5 | H6 |
| --- | --- | --- | --- | --- | --- | --- |
|  | C1 | C2 | C3 | C4 | C5 | C6 |
| A | 5.02 | 3.92 | 4.35 | 4.35 | 3.88 | — |
|  | 100.4 | 71.1 | 79.5 | 70.2 | 72.8 | 170.8 |
| B | 4.51 | 3.98 | 4.38 | 4.75 | 3.83 | 3.92 |
|  | 96.0 | 71.2 | 79.5 | 70.7 | 72.9 | 68.0 |
| C | 4.53 | 3.98 | 4.27 | 3.93 | 3.66 | 3.69 |
|  | 95.3 | 71.2 | 78.8 | 68.4 | 70.1 | 67.9 |
| D | 5.25 | 4.75 | 3.67 | 4.20 | 3.88 | 1.22 |
|  | 102.8 | 70.3 | 72.4 | 71.9 | 65.6 | 16.5 |
| E | 5.25 | 4.75 | 3.67 | 4.17 | 3.84 | 1.22 |
|  | 102.8 | 70.3 | 72.4 | 70.0 | 65.1 | 16.3 |
| F | 5.41 | 3.85 | 3.75 | 4.00 | 3.52 |  |
|  | 106.9 | 78.5 | 72.4 | 68.7 | 60.4 |  |
| G | 5.43 | 3.89 | 3.76 | 4.03 | 3.63 |  |
|  | 107.1 | 78.6 | 72.5 | 69.7 | 65.8 |  |
